# Obesity-associated non-oxidative genotoxic stress alters trophoblast turnover in human first-trimester placentas

**DOI:** 10.1093/molehr/gaae027

**Published:** 2024-08-02

**Authors:** Denise Hoch, Alejandro Majali-Martinez, Julia Bandres-Meriz, Martina Bachbauer, Caroline Pöchlauer, Theresa Kaudela, Ezgi Eyluel Bankoglu, Helga Stopper, Andreas Glasner, Sylvie Hauguel-De Mouzon, Martin Gauster, Silvija Tokic, Gernot Desoye

**Affiliations:** Department of Obstetrics and Gynaecology, Medical University of Graz, Graz, Austria; Department of Obstetrics and Gynaecology, Medical University of Graz, Graz, Austria; Departamento de Medicina, Facultad de Ciencias Biomédicas y de la Salud, Universidad Europea de Madrid, Madrid, Spain; Department of Obstetrics and Gynaecology, Medical University of Graz, Graz, Austria; Department of Obstetrics and Gynaecology, Medical University of Graz, Graz, Austria; Department of Obstetrics and Gynaecology, Medical University of Graz, Graz, Austria; Department of Obstetrics and Gynaecology, Medical University of Graz, Graz, Austria; Institute of Pharmacology and Toxicology, University of Wuerzburg, Wuerzburg, Germany; Institute of Pharmacology and Toxicology, University of Wuerzburg, Wuerzburg, Germany; Femina Med Center, Graz, Austria; Department of Reproductive Biology, Case Western Reserve University, Cleveland, OH, USA; Division of Cell Biology, Histology and Embryology, Gottfried Schatz Research Center, Medical University of Graz, Graz, Austria; Department of Paediatrics and Adolescent Medicine, Medical University of Graz, Graz, Austria; Research Unit of Analytical Mass Spectrometry, Cell Biology and Biochemistry of Inborn Errors of Metabolism, Medical University of Graz, Graz, Austria; Department of Obstetrics and Gynaecology, Medical University of Graz, Graz, Austria

**Keywords:** placental growth, trophoblast, proliferation, apoptosis, senescence, early pregnancy, DNA damage response

## Abstract

Placental growth is most rapid during the first trimester (FT) of pregnancy, making it vulnerable to metabolic and endocrine influences. Obesity, with its inflammatory and oxidative stress, can cause cellular damage. We hypothesized that maternal obesity increases DNA damage in the FT placenta, affecting DNA damage response and trophoblast turnover. Examining placental tissue from lean and obese non-smoking women (4–12 gestational weeks), we observed higher overall DNA damage in obesity (COMET assay). Specifically, DNA double-strand breaks were found in villous cytotrophoblasts (vCTB; semi-quantitative γH2AX immunostaining), while oxidative DNA modifications (8-hydroxydeoxyguanosine; FPG-COMET assay) were absent. Increased DNA damage in obese FT placentas did not correlate with enhanced DNA damage sensing and repair. Indeed, obesity led to reduced expression of multiple DNA repair genes (mRNA array), which were further shown to be influenced by inflammation through *in vitro* experiments using tumor necrosis factor-α treatment on FT chorionic villous explants. Tissue changes included elevated vCTB apoptosis (TUNEL assay; caspase-cleaved cytokeratin 18), but unchanged senescence (p16) and reduced proliferation (Ki67) of vCTB, the main driver of FT placental growth. Overall, obesity is linked to heightened non-oxidative DNA damage in FT placentas, negatively affecting trophoblast growth and potentially leading to temporary reduction in early fetal growth.

## Introduction

Obesity prevalence has increased in women of reproductive age. In the USA, almost 35% of pregnant women are obese (Chen *et al.*, 2018), which carries short- and long-term risks for mother and offspring (Poston *et al.*, 2016). The placenta, a fetal organ, is positioned between the maternal and fetal circulation and fulfills a range of functions to sustain adequate fetal development (Desoye, 2018). It is exposed to maternal blood through an outer tissue layer, the syncytiotrophoblast (STB). This multinucleated syncytium is formed by fusion with subjacent, proliferating villous cytotrophoblast (vCTB) (Aplin and Jones, 2021). STB renewal by continuous fusion with proliferating vCTB is highly regulated in both a spatial and time-dependent manner to ensure proper placental surface expansion and placental growth (Aplin and Jones, 2021).

Trophoblast turnover, and hence, placental development, is strongly influenced by the maternal metabolic and endocrine environment (Hoch *et al.*, 2019). In the first trimester (FT) of pregnancy, the placental growth rate, mostly dictated by vCTB proliferation, is highest (Desoye, 2018), while DNA methylation levels are lowest in comparison to later stages of pregnancy (Novakovic *et al.*, 2011). Rapidly proliferating tissues are vulnerable to environmental influences and DNA methylation is important for maintaining genome integrity (Sriraman *et al.*, 2020). Sensitivity of FT placenta to environmental perturbations was established for various pregnancy conditions (Lassance *et al.*, 2015a; Hoch *et al.*, 2023) including Type 1 diabetes (Hiden *et al.*, 2008; Gauster *et al.*, 2017; Majali-Martinez *et al.*, 2021). Increased production of pro-inflammatory cytokines with accompanying meta-inflammatory stress are hallmarks of obesity (Hotamisligil, 2006). Moreover, oxygen tension and its physiological changes in the FT are powerful regulators of vCTB proliferation and placental development (Jauniaux *et al.*, 2000; Burton *et al.*, 2010). Thus, also obesity-associated changes in oxygen tension may influence placental development and function (Hoch *et al.*, 2019).

Recently, we identified a network of cell cycle regulators affected by maternal obesity in FT placental tissue with upregulated breast cancer 1 (BRCA1) present predominantly in vCTB (Hoch *et al.*, 2020a). This may reflect the high proliferative potential of vCTB requiring adequate cell cycle control. Proper activation of cell cycle checkpoints ensures genome stability by preventing damaged cells from entering mitosis and by activating DNA damage repair pathways (Barzilai and Yamamoto, 2004). Accordingly, BRCA1 upregulation by maternal obesity might indicate compromised DNA integrity.

DNA integrity is maintained by adequate DNA damage repair pathways in response to specific forms of DNA damage, e.g. oxidative DNA damage, single (SSB) or double-strand break (DSB). A central player in maintaining DNA integrity by sensing DNA damage is ataxia telangiectasia mutated (ATM) (Marechal and Zou, 2013). The cells respond to detected damage first by increasing S139 phosphorylation of H2A histone family member X (γH2AX) (Stiff *et al.*, 2004; Plesca *et al.*, 2008; Blackford and Jackson, 2017). Second, ATM binds to ataxia telangiectasia and Rad3-related (ATR), forming ATM/ATR complexes, which bind to damaged sites and target downstream effectors, including BRCA1, Chk2, and p53 (Blackford and Jackson, 2017). These are involved in multiple cellular downstream processes like cell cycle transition, DNA repair, gene transcription, protein translation, and degradation, which can ultimately lead to apoptosis (Barzilai and Yamamoto, 2004) and consequently affect cellular turnover.

Here, we hypothesized that maternal obesity is associated with genotoxic stress reflected by enhanced DNA damage in the vCTB already in FT of human pregnancy. We have focused on deciphering type and cellular location of DNA damage and on identifying affected downstream targets involved in DNA damage sensing and repair. Moreover, we studied the consequences of DNA damage on fundamental processes determining trophoblast turnover, i.e. proliferation, apoptosis, and senescence.

## Materials and methods

### Ethical approval

The study was approved by the institutional review board and ethical committee of Medical University of Graz (29-095 ex16/17) and was performed in accordance with the latest revision of the Declaration of Helsinki. Women with a singleton pregnancy scheduled for legal elective pregnancy termination were recruited upon signing written informed consent. Patient details were limited to those listed in Supplementary Table S1 and below (c.f. Tissue collection) to protect patient privacy as a requirement of the ethics approval.

### Tissue collection

FT placental tissue (gestational week 5–12 post menstruation) was obtained after voluntary termination (total n = 101, specific numbers are stated for each experiment). Exclusion criteria were smoking assessed by questionnaire and verified by serum cotinine concentrations using a cut-off >4.47 ng/ml (Benowitz *et al.*, 2009), other co-morbidities, and current medication. BMI, calculated from body weight (kg) measured at the time of pregnancy termination and height (m), ranged from 19.2 to 39.5 kg/m^2^. Gestational age was calculated based on women’s last menstrual period and verified by measurement of fetal crown–rump length. Cases with gestational age difference between last menstrual period and fetal crown–rump length >6 days were excluded. Tissue was washed with phosphate-buffered saline (PBS, Sigma Aldrich, St Louis, MO, USA) and cryopreserved at −80°C after snap-freezing or formalin-fixed and paraffin-embedded until further use. Time between tissue collection and cryopreservation was recorded (‘preparation time’) and used as a covariate (Cindrova-Davies *et al.*, 2015).

### FT chorionic villus explant culture

Human FT chorionic villi were micro-dissected (15–20 mg wet weight), rinsed with PBS, and cultured in Dulbecco’s Modified Eagle Medium (Gibco, Invitrogen, Carlsbad, CA, USA) and Ham’s F-12 medium 1:1 (v/v; Gibco) supplemented with 10% (v/v) fetal calf serum (Thermo Scientific, Rockford, IL, USA) and 1% (v/v) penicillin–streptomycin (Gibco). Explants were pre-conditioned at 2.5% O_2_ in a hypoxic workstation (BioSpherix, Redfeld, NY, USA) for 24 h, treated with tumor necrosis factor (TNF)-α (50 ng/ml, Sigma Aldrich) at 2.5% O_2_ for 48 h, and snap-frozen for subsequent RNA extraction or protein isolation. Culture supernatants were frozen and used for human β-chorionic gonadotropin quantification (IMMULITE 1000 Systems Immunoassay, Siemens, Munich, Germany).

### Alkaline and enzyme-modified (formamidopyrimidine DNA glycosylase) COMET assay

FT placental tissue was minced with cold Merchant medium and filtered through a 20-µm sieve. After washing with PBS, cells were centrifuged at 400 *g*, 5 min, 4°C, re-suspended in cold Merchant medium, and mixed with preheated 0.8% (w/v) low melting point agarose. This mixture was placed on agarose-precoated slides and kept on ice. After solidification, slides were placed in lysis solution (2.5 M NaCl, 10 mM EDTA, 10 mM Tris, 1% (v/v) Triton X-100, 5% (v/v) dimethylsulfoxide) for 1 h. Slides for the *ex vivo* H_2_O_2_ challenge were immersed in cold H_2_O_2_ solution (50 µM, 5 min, 4°C) prior to lysis. Afterwards, slides were washed 3× for 5 min with enzyme reaction buffer (40 mM HEPES, 0.1 M KCl, 0.5 mM EDTA, 0.2 mg/ml bovine serum albumin, pH 8.0) and incubated either with formamidopyrimidine DNA glycosylase (FPG) enzyme or with enzyme reaction buffer for 1 h at 37°C. All slides were placed in cold alkaline buffer (1 mM EDTA, 300 mM NaOH, pH ≥13) in an electrophoresis chamber, 20 min, in the dark to allow unwinding of DNA before electrophoresis (∼1 V/cm, 20 min on ice). Thereafter, slides were immersed in PBS and bi-distilled water for 10 min and fixed in 70% followed by 100% ethanol for 15 min. After air-drying, slides were stained with GelRed (Sigma Aldrich) and 100 nuclei were scored per subject by using Komet 6 software (BFI Optilas, Puchheim, Germany). The net FPG-sensitive sites in the respective sample were quantified by subtracting the percentage of DNA in tail in buffer controls.

### DNA/RNA isolation and reverse transcription

FT placental tissue was homogenized in RLT Plus Buffer (Qiagen, Hilden, Germany) with 1% (v/v) β-mercaptoethanol (Merck, Darmstadt, Germany) using a tissue lyser (MagNa Lyser, Roche, Basel, Switzerland). DNA and total RNA were isolated with the AllPrep DNA/RNA/miRNA Universal Kit (Qiagen) according to manufacturer’s guidelines. mRNA quality was controlled using Bioanalyzer (Agilent, Santa Clara, CA, USA) with an inclusion cut-off RNA integrity number (RIN) ≥3. Three samples with RIN <3 were not included in further analyses. RIN values (mean ± SD) of all samples for Nanostring analyses were 7.1 ± 1.0. After quality control, mRNA was reverse transcribed using SuperScript II Reverse Transcriptase kit (Life Technologies, Carlsbad, CA, USA) as per the manufacturer’s protocol.

### Nanostring

Gene expression was quantified using NanoString nCounter system (Nanostring Technologies, Seattle WA, USA), which is based on the digital detection of mRNA molecules using color-coded probe pairs that specifically hybridize to target molecules. Gene expression was measured by counting the barcode for each specific molecule, which is detected by digital analyzer. Probe pairs were synthesized at Integrated DNA Technologies (Leuven, Belgium). Positive normalization to the geo-mean of top three positive controls and codeset normalization (Molania *et al.*, 2019) to reference genes WD Repeat Domain 45B (WDR45L) and TATA-binding protein were performed by nSolver 4.0 software (Nanostring Technologies). Results are expressed as gene counts of mRNA molecules in 100 ng/µl RNA.

### Protein isolation, quantification, and immunoblotting

FT placental tissue was homogenized in RIPA buffer (Sigma Aldrich) with protease inhibitors (Roche, Basel, Switzerland). Protein amount was quantified using a BCA assay (Thermo Scientific). Protein lysates were mixed with Laemmli buffer 2× (Sigma Aldrich) and denatured for 5 min at 96°C. Total protein (10 µg) was loaded onto 4–20% SDS-PAGE gels (BioRad), separated at 120 V for 1 h, and transferred onto nitrocellulose membranes using a TurboBlot system (BioRad). Blocking was performed for 1 h with 5% non-fat dry milk (BioRad) in Tris-borate-EDTA +0.1% Tween 20 (Sigma Aldrich). Membranes were incubated with primary antibodies (Table 1) overnight at 4°C. Thereafter, membranes were incubated with horseradish peroxidase-conjugated secondary antibody (1:2000, BioRad), 1 h at room temperature. Immunoreactivity using SuperSignal-Pico Chemiluminescent Substrate (Thermo Scientific) was visualized with a Fusion FX system (Vilber Lourmat, Eberhardzell, Germany). Band intensities were quantified using EvolutionCapt software (Vilber Lourmat, Eberhardzell, Germany). Results are presented as a ratio of band densities of target protein and reference protein.

**Table 1.** Antibodies used for immunoblotting and immunofluorescence.

Immunoblotting			
Antigen	Species	Dilution	Source (catalog number)
HSP70	Mouse	1:1000	Invitrogen, Carlsbad, CA, USA (MA3-006)
HO-1	Rabbit	1:2000	EnzoLife Science, Farmingdale, NY, USA (ADI-SPA-895)
ATM	Rabbit	1:1000	Cell Signaling Technology, Danvers, MA, USA (#2873)
ATR	Rabbit	1:1000	Merck, Darmstadt, Germany (09-070)
p-ATM/ATR Substrates (S*Q)	Rabbit	1:1000	Cell Signaling Technology, Danvers, MA, USA (#9607)
P53	Mouse	1:500	Santa Cruz Biotechnology, Dallas, TX, USA (sc-126)
p^Ser15^-p53	Rabbit	1:1000	Cell Signaling Technology, Danvers, MA, USA (#9284)
P16	Rabbit	1:1000	Abcam, Cambridge, UK (ab108349)
β-actin	Mouse	1:10,000	Abcam, Cambridge, UK (ab8227)
α-tubulin	Mouse	1:1000	Merck, Darmstadt, Germany (CP06-100UG)

Immunofluorescence			
Antigen	Species	Dilution	Source, catalog number
E-cadherin	Mouse	1:100	Cell Signaling Technology, Danvers, MA, USA (#14472)
γH2AX	Rabbit	1:100	Merck, Darmstadt, Germany (05-636NA-1KL)
Caspase-cleaved cytokeratin 18	Rabbit	1:25	Roche, Basel, Switzerland (14533800)
Ki-67	Rabbit	1:100	Thermo Scientific, Rockford, IL, USA (RM-9106-S0)
Alexa Fluor 568 anti-mouse IgG	Goat	1:2000	Thermo Scientific, Rockford, IL, USA (11004)
Alexa Fluor 488 anti-mouse IgG	Goat	1:2000	Thermo Scientific, Rockford, IL, USA (11001)
Alexa Fluor 568 anti-rabbit IgG	Goat	1:2000	Thermo Scientific, Rockford, IL, USA (11011)
Alexa Fluor 488 anti-rabbit IgG	Goat	1:2000	Thermo Scientific, Rockford, IL, USA (11008)

HSP70, heat shock protein 70; HO-1, heme-oxygenase 1; γH2AX, H2A histone family member X.

### Oxidative DNA damage ELISA

Levels of 8-hydroxydeoxyguanosine (8-OHdG) were measured in DNA extracted from FT villous tissues using an OxiSelect Oxidative DNA Damage ELISA Kit (Cell Biolabs, Heidelberg, Germany) according to the manufacturer’s protocol. DNA was converted to single-stranded DNA by incubation, 5 min, 95°C, followed by digestion to nucleosides by nuclease P1 incubation, 2 h, 37°C. Samples were incubated with an 8-OHdG antibody, 1 h, room temperature. Absorbance (450 nm) was measured with SPECTROstar Nano (BMG Labtech, Ortenberg, Germany).

### Immunofluorescence

Formalin-fixed and paraffin-embedded placental tissue sections (3 µm) were placed on Superfrost Plus slides (Menzel, Thermo Scientific). Following standard deparaffinization, heat-induced antigen retrieval was performed in citrate buffer (pH 6, Merck) in a Decloaking Chamber (Biocare Medical, Pacheco, CA, USA), 15 min, 120°C.

Cell death was detected *in situ* by TUNEL (TdT-mediated dUTP-biotin nick end labeling) assay as per the manufacturer’s protocol (#11684795910, Roche Applied Science, Basel, Switzerland). FT placental tissue exposed to X-ray (dose-length product: 6889.6 mGy.cm) served as a positive control.

Immunofluorescence was performed as previously described (Fuchs *et al.*, 2021) using primary and secondary antibodies listed in Table 1. Negative control slides were incubated with rabbit or mouse IgG fractions (1:100, Dako) as isotype controls. Slides were mounted with ProLong Gold Antifade Mountant with DAPI (Thermo Scientific). Images were captured using an LSM 510 META Axiovert 200M confocal system with a 40× Plan-Neofluar 1.3 DIC oil immersion objective and ZEN 2012 software (all Carl Zeiss Meditec AG, Jena, Germany). Analysis of TUNEL/cytokeratin 18 (CK18)/Ki67 positive nuclei and γH2AX foci in trophoblast cells was performed using ImageJ (ImageJ, National Institutes of Health, New York, NY, USA).

### Fetal sex determination

Fetal sex was determined for a subset of samples (73% of total samples) by assessing *XIST* and *DDX3Y* gene expression by RT-qPCR using FAM- and VIC-labeled TaqMan gene expression assays (Life Technologies, XIST: Hs01079824_m1; DDX3Y: Hs00965254_gH), TaqMan universal PCR master mix (Life Technologies), and the CFX96 and CFX384 real-time PCR detection systems (BioRad Laboratories, Hercules, CA, USA) as described previously (Hoch *et al.*, 2020b).

### Statistical analysis

For statistical analysis, IBM SPSS Statistics 25 (Armonk, NY, USA) and GraphPad Prism 8 (Boston, MA, USA) were used. Normal distribution of data was tested using the Shapiro–Wilk test. Associations between maternal BMI and experimental outcomes were assessed using multivariate linear regression models with adjustments for fetal sex and the predefined confounders gestational age and preparation time. Gestational age and preparation time were considered as continuous variables, while maternal BMI was either categorized into lean (BMI <25 kg/m^2^) and obese (BMI ≥30 kg/m^2^) or used as a continuous variable. Where possible, in additional subanalyses, data were stratified according to fetal sex (alkaline COMET assay, Immunoblots, Nanostring, caspase-cleaved CK18 and p21 immunoblot) and analyzed by multivariate ANOVA with maternal BMI and fetal sex as fixed variables and gestational age and preparation time as covariates. As determined by multivariate analysis, maternal age had no effect on results and was not included in the final model as a confounder. A *P* < 0.05 was considered statistically significant.

## Results

### Maternal obesity is associated with FT placental DNA damage

**Figure 1.**
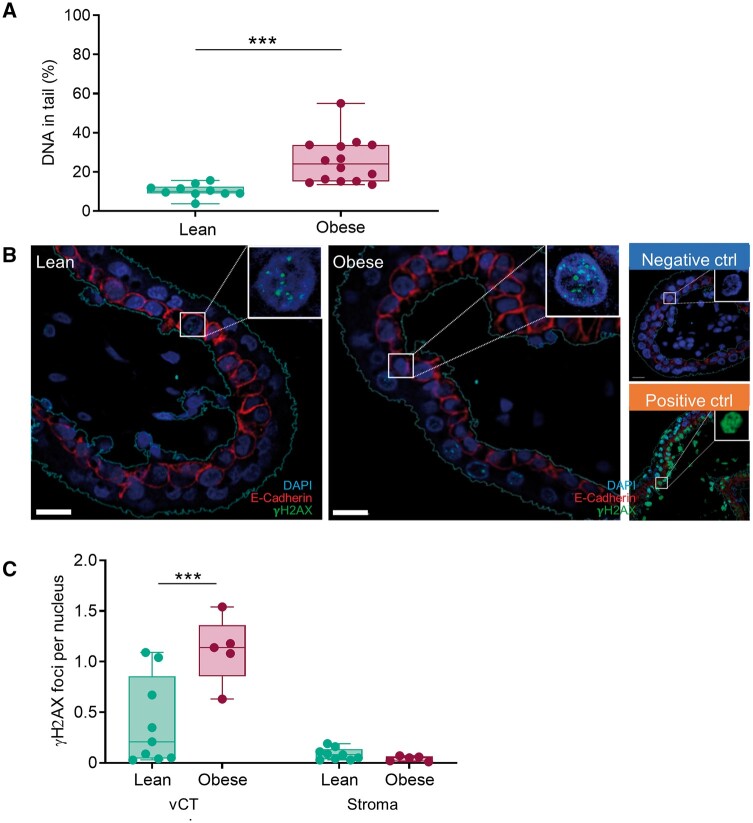
**Obesity increases DNA damage in first trimester (FT) tissue.** (**A**) Alkaline COMET assay was used to assess overall DNA damage in total placental tissue from lean (BMI <25, n = 10) and obese (BMI >30, n = 14) women. (**B**) Immunofluorescence double staining for the DNA DSB marker γH2AX (green) and E-Cadherin (red) to demarcate vCT from stromal cells in FT placental sections from lean (n = 9) and obese (n = 5) women. (**C**) Quantification of immunofluorescence double staining by ImageJ for trophoblast and stromal cells, respectively. For (A–C), statistical analysis was performed using a multivariate linear regression analysis with BMI as categorical dependent variable and data were adjusted for gestational age, processing time, and fetal sex. Fetal sex had no effect (*P* = 0.439) in the alkaline COMET assay as tested by multivariate ANOVA with fetal sex and BMI as categorical fixed variables and gestational age and processing time as covariates. Results are presented as mean ± SD. (A) gestational week 5–12, (B) and (C) gestational week 5–7. ***P < 0.001. BMI, body mass index; vCT, villous cytotrophoblasts; γH2AX, H2A histone family member X.

To investigate whether obesity is associated with genomic DNA damage in the FT placenta, we performed an alkaline COMET assay using total placental tissue from lean and obese women. This assay detects various forms of DNA damage: DNA SSB and DSB, alkali-labile sites, DNA–DNA/DNA–protein cross-linking, and incomplete excision repair sites (Pu *et al.*, 2015). In FT placental tissue of obese women, DNA damage was increased by 2.6-fold compared to lean controls (*P* < 0.001, Fig. 1A). This was initially analyzed for 5–7 and 6–9 weeks, respectively, and then expanded to the whole range, i.e. 5–12 weeks, without significant differences in the results.

### Maternal obesity is associated with DNA DSB in vCTB

To identify type and cellular location of DNA damage, a double immunofluorescence staining for γH2AX, a DNA DSB marker, and for E-Cadherin, to demarcate vCTB, was performed in FT placental tissue of lean and obese women. Only vCTB of placentas from obese women showed an increase (6.9-fold, *P* = 0.001) in γH2AX foci reflecting DNA DSB (Fig. 1B), while non-trophoblast cells remained unaffected as compared to lean controls (Fig. 1C).

### Maternal obesity is not associated with placental oxidative stress-induced DNA damage in FT

**Figure 2.**
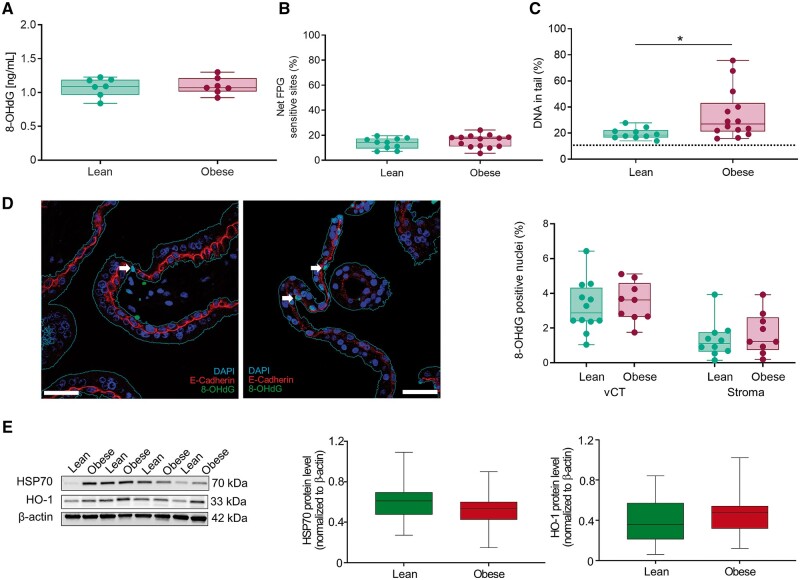
**Oxidative stress is not the culprit for obesity-associated DNA damage in first trimester (FT) trophoblasts.** (**A**) Levels of 8-hydroxydeoxyguanosine (8-OHdG) measured by ELISA in tissues from lean (n = 7) versus obese (n = 7) women at 7 weeks of gestation in duplicates. Statistics were performed by an unpaired Student’s *t*-test. Data are shown as mean ± SD. (**B**) Alkaline COMET assay with formamidopyrimidine DNA glycosylase (FPG) modification was used to determine oxygen-induced DNA stand breaks in total placental tissue from lean and obese women. (**C**) Placental tissue treated with H_2_O_2_ prior to alkaline COMET assay served as a positive control. Dotted line indicates basal DNA in tail in placental tissue of lean women without H_2_O_2_ treatment (10.42%). For (B) and (C), statistical analysis was performed using a multivariable linear regression analysis with BMI as categorical dependent variable and data were adjusted for gestational age and processing time. Data shown as mean ± SD. n (lean/obese) = 10/14. Gestational week 5–12. *P < 0.05. (**D**) Detection of 8-OHdG in paraffin-embedded FT placenta sections by double immunofluorescence staining for 8-OHdG and E-cadherin. Statistical analysis was performed using a multivariable linear regression analysis with BMI as categorical dependent variable and data were adjusted for gestational age and processing time. Results are presented as mean ± SD. n (lean/obese) = 12/9. Gestational week 5–12. White arrow indicates positive nuclei. (**E**) Immunoblots of HSP70 and HO-1 in FT placental tissue were quantified by densitometric analysis. β-actin was used as loading control for normalization. Statistical analysis was performed using a multivariable linear regression analysis with BMI as categorical dependent variable and data were adjusted for gestational age, processing time and fetal sex. Fetal sex had no effect (*P* = 0.997) in the alkaline COMET assay with FPG modification, when tested by multivariate ANOVA with fetal sex and BMI as categorical fixed variables and gestational age and processing time as covariates. Results are presented as mean ± SD. n (lean/obese) = 18/18. Gestational week 5–12. BMI, body mass index; vCT, villous cytotrophoblasts; HSP70, heat shock protein 70; HO-1, heme-oxygenase 1; 8-OHdG, 8-hydroxydeoxyguanosine.

Oxidative stress is a hallmark of obesity. Therefore, we sought evidence for excessive oxidative stress. We quantified placental 8-OHdG, the product of oxygen-free radical-induced guanosine oxidation, by ELISA at 7 weeks. We extended the gestational age range to 5–12 weeks and confirmed the results by FPG COMET assay. Placental tissue from lean and obese women did not differ in 8-OHdG levels (Fig. 2A) or net FPG-sensitive sites (Fig. 2B). We demonstrated the ability of oxidative stress to induce DNA damage in FT placental tissue by an *in vitro* H_2_O_2_ challenge prior to the alkaline COMET assay (Fig. 2C).

Analyses of total placental tissue may mask cell type-specific alterations in DNA damage such as DSBs, which are present in higher quantity only in the trophoblast compartment (Fig. 1C). Therefore, we quantified trophoblast and stroma-specific nuclear 8-OHdG content by double immunofluorescence staining for 8-OHdG and E-Cadherin. Maternal obesity did not alter oxidative DNA damage neither in FT trophoblasts nor in stromal cells (Fig. 2D).

Absence of oxidative DNA damage raised the question whether the FT placenta is under oxidative stress at all. Thus, we quantified by immunoblotting heat shock protein 70 (HSP70) and heme-oxygenase 1 (HO-1) protein as stress markers. No differences in both HSP70 and HO-1 protein levels were found between FT placental tissue from lean and obese women (Fig. 2E and Supplementary Fig. S1).

These results argue for absence of excessive oxidative stress in the FT placenta of obese women to account for the increased DNA damage in these samples.

### Impaired DNA damage sensing in FT placentas of obese women

**Figure 3.**
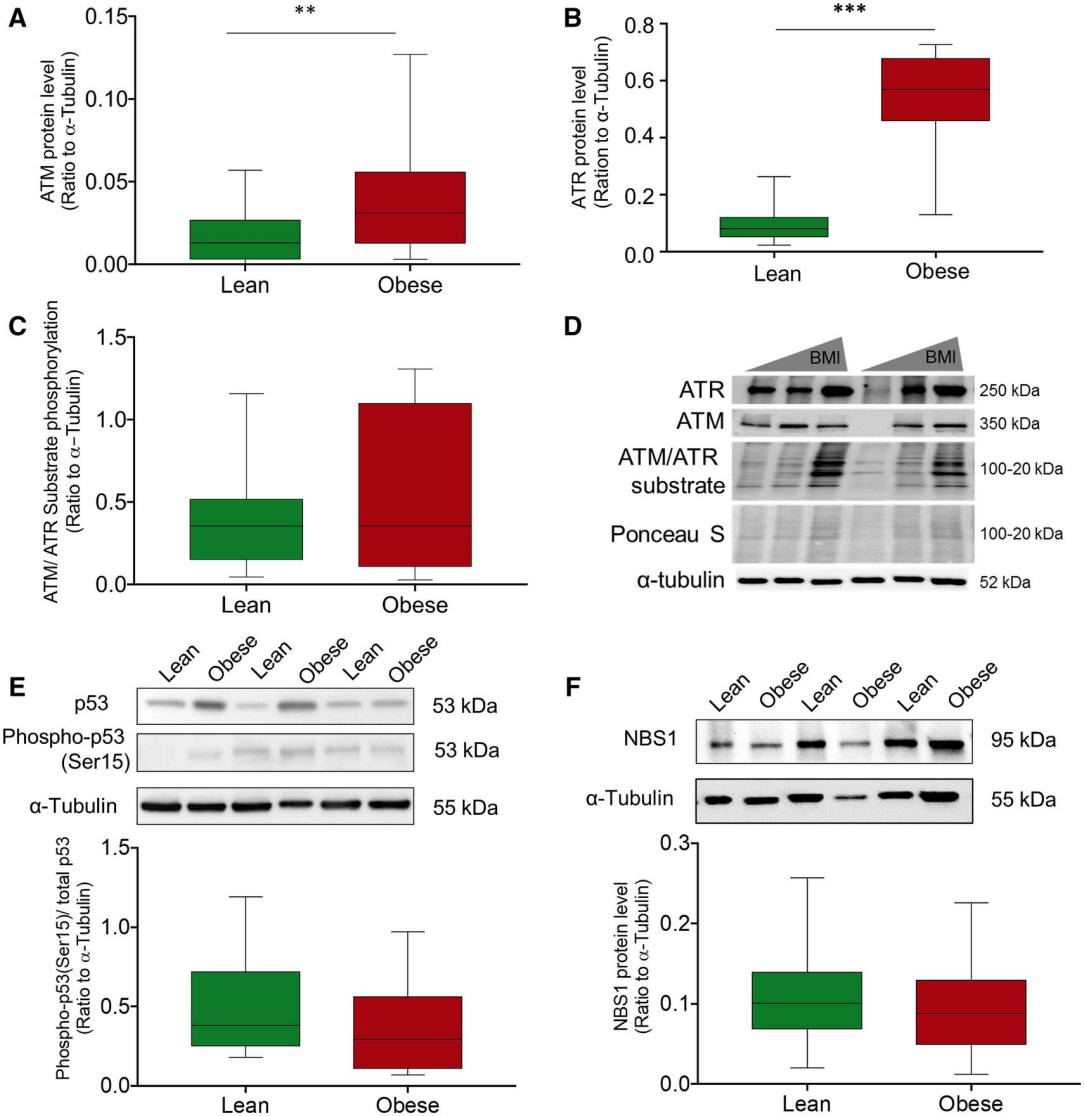
**DNA damage sensing and downstream signaling in first trimester (FT) placentas of lean and obese women.** (**A**) ATM and (**B**) ATR total protein quantification by immunoblot analysis and (**C**) ATM/ATR substrate phosphorylation indicating main downstream targets of ATM/ATR complex. (**D**) Representative immunoblots for ATM, ATR, and ATM/ATR substrates (**E**) and (**F**) ATM/ATR downstream effectors phospho-p53/total p53 and NBS1, respectively, in FT placenta of lean and obese women. From (A)–(F), statistical analysis was performed using a multivariate linear regression analysis with BMI as categorical dependent variable and data were adjusted for gestational age and processing time. The relative density ratio of each protein was calculated to α-tubulin. Data are shown as means ± SD, n (lean/obese) = 18/18. Gestational week 5–12. ** *P* < 0.01; *** *P* < 0.001. ATM, ataxia telangiectasia mutated; ATR, ataxia telangiectasia and Rad3-related; BMI, body mass index.

ATM/ATR signaling is central to the regulatory network sensing DNA lesions with consequences for expression of genes involved in damage repair, cell cycle arrest, and apoptosis. To determine whether the tissue responded adequately to the observed increased DNA damage, we assessed ATM and ATR substrate phosphorylation of serines/threonines in S*/T*-Q motif (Kim *et al.*, 1999). Maternal obesity increased placental ATM (2.8-fold, *P* < 0.01) (Fig. 3A and D) and ATR (5.9-fold, *P* < 0.001) (Fig. 3B and D) protein, but total ATM/ATR substrate phosphorylation was not altered (Fig. 3C and D). Unmodified western blot images for Fig. 3D are shown in Supplementary Fig. S2.

ATM/ATR-dependent phosphorylation leads to stabilization and activation of p53 via phosphorylation at SER^15^, the classical ATM phosphorylation site (Blackford and Jackson, 2017). Similar to obese adipocytes (Vergoni *et al.*, 2016), total p53 protein level was increased by trend (*P* = 0.07) in FT placental tissue from obese women and total SER^15^-p53 phosphorylation was also increased, but unchanged relative to p53 (Fig. 3E and Supplementary Fig. S3). Ninbrin (NBS1), part of the sensor protein complex recruiting predominantly ATM to damage sites (Zhou *et al.*, 2006), was unaltered (Fig. 3F and Supplementary Fig. S3). Collectively, these results suggest a problem with activation of ATM/ATR-mediated DNA damage response in obesity.

### Impaired placental DNA damage repair in the FT of obese women

After DNA damage recognition, a cascade of highly regulated proteins involved in damage repair is activated. To analyze the obesity effect on the complex DNA damage repair machinery, we quantified expression of 21 genes involved in DNA damage repair signaling pre-selected based on the literature.

**Figure 4.**
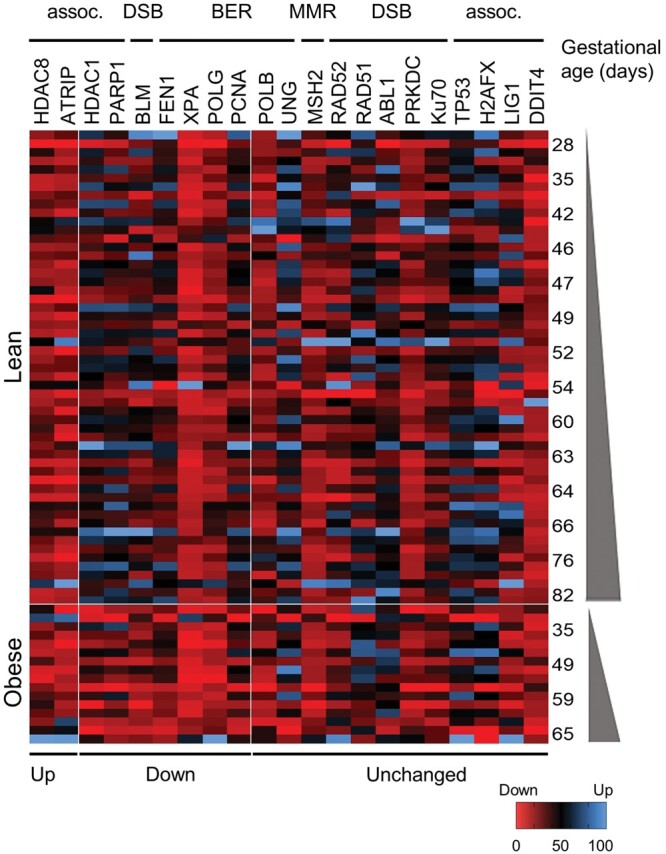
**Heat map illustrating the effect of maternal obesity on first trimester (FT) placental genes involved in the DNA repair.** Gene expression was determined in FT placental tissue from lean and obese women by NanoString analysis. Blue indicates upregulation and red indicates downregulation by obesity, respectively. Gene counts were normalized to the mean of two different housekeeping genes WD repeat domain 45B (WDR45L) and TATA-box-binding protein (TBP). Genes were grouped according to the DNA repair pathway they are involved in: assoc, genes associated with DNA repair pathways; DSB, double-strand break; BER, base excision repair; MMR, mismatch repair. Statistical analysis was performed using a multivariate linear regression analysis with BMI as categorical dependent variable adjusted for gestational age and tissue processing time. In a multivariate ANOVA with fetal sex and BMI as categorical fixed variables and gestational age and processing time as covariates, fetal sex influenced expression of *HDAC8* (*P* < 0.004), *XPA* (*P* = 0.006), and *ATRIP* (*P* = 0.021). n (lean/obese) = 54/18. Gestational week 5–12 (28–84 days). BMI, body mass index.

Among the 10 genes affected by obesity, 7 were expressed at lower levels potentially indicating a decrease in DNA repair capacity: *HDAC1* (−20.0%, *P* = 0.01), *PARP1* (−23.6%, *P* = 0.02), *BLM* (−35.2%, *P* < 0.023), *FEN1* (−34.0%, *P* = 0.013), *XPA* (−4.8%, *P* < 0.001), and *POLG* (−7.8%, *P* = 0.04). *PCNA*, an important gene in DNA damage response, was reduced by trend (−20%, *P* = 0.08). Only histone deacetylase-8 (*HDAC8*) and ATR interacting protein (*ATRIP*) expression were upregulated by 9.2% (*P* < 0.001) and 16.2% (*P* = 0.025), respectively, in FT placental tissue of obese women. Grouping of expression data based on the various DNA damage repair signaling pathways identified key transducers in both SSB encompassing base excision repair (BER) and mismatch repair and in DSB repair (Fig. 4).

**Figure 5.**
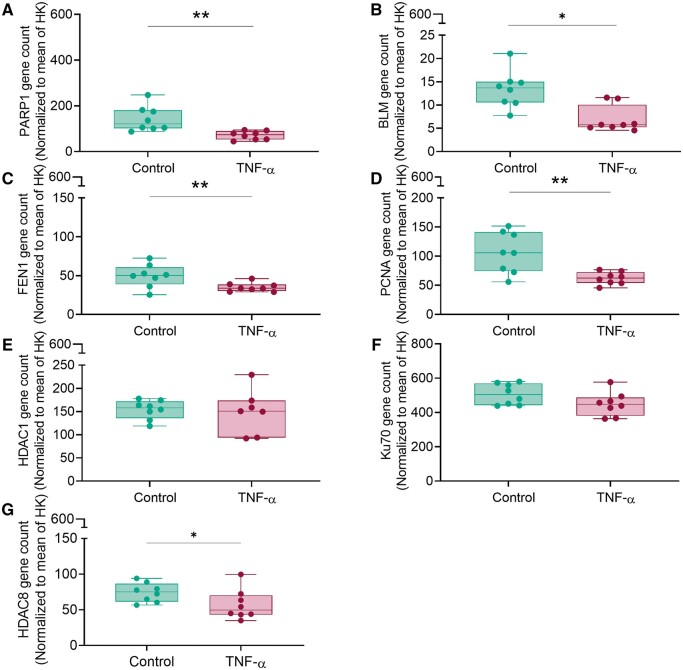
**Effect of inflammation on expression of DNA damage repair genes in first trimester (FT) placental explants.** FT chorionic villous explants of lean women were cultured at 2.5% O_2_ with or without TNF-α (50 ng/ml) for 48 h in triplicates. Gene expression of PARP1 (**A**), BLM (**B**), FEN1 (**C**), PCNA (**D**), HDAC1 (**E**), Ku70 (**F**), and HDAC8 (**G**), which were regulated by maternal obesity (Fig. 4), was determined by Nanostring analysis. Data were normalized to the mean of two different housekeeping (HK) genes WD repeat domain 45B (WDR45L) and TATA box binding protein (TBP). Statistical analysis included paired *t*-test or Wilcoxon matched-pairs signed rank test. Results are presented as mean ± SD of n = 8 different explants measured in triplicates. Gestational week 5–10. * *P* < 0.05, ** *P* < 0.01. TNF, tumor necrosis factor.

Since we found no evidence for more oxidative stress in obesity, we tested the potential of inflammatory stress to induce changes in DNA damage repair genes. To this end, we used FT chorionic villous explants (gestational age 5–10 weeks) and treated the tissue at the physiological oxygen tension of 2.5% oxygen with TNF-α (50 ng/ml), the classical pro-inflammatory cytokine. Tissue viability was unaffected because human β-chorionic gonadotropin concentrations in culture supernatant were unaltered (data not shown) (Polliotti *et al.*, 1995). TNF-α downregulated some of the DNA damage repair genes, which are also altered by obesity: *PARP1* (−49.3%, *P* = 0.008), *BLM* (−46.9%, *P* = 0.02), *FEN1* (−25.5%, *P* = 0.04), and *PCNA* (−38.0%, *P* = 0.008) (Fig. 5A–D), but did not affect *HDAC1* and *Ku70* gene expression (Fig. 5E and F). This data suggest obesity-associated inflammation as a potential candidate accounting for some of the effects found in obesity. However, as TNF-α induced downregulation of *HDAC8* (−24.0%, *P* = 0.04) (Fig. 5G) in explants vis-à-vis, its upregulation in tissue of obese women demonstrates a more complex regulation. It may also reflect response differences to acute, i.e. *in vitro*, versus chronic, i.e. *in vivo*, exposure to the obesity-associated environment.

### Consequences for FT trophoblast turnover in obesity

Unrepaired DNA damage directly affects cellular turnover by reducing proliferation rate and/or initiation of apoptotic events. Increased trophoblast DNA damage and inadequate cellular DNA repair response raised the question about potential consequences for vCTB turnover. We assessed trophoblast-specific viability by measuring proliferation and apoptosis *in situ* and measured p16 protein levels to determine cellular senescence as a further potential obesity-induced consequence.

**Figure 6.**
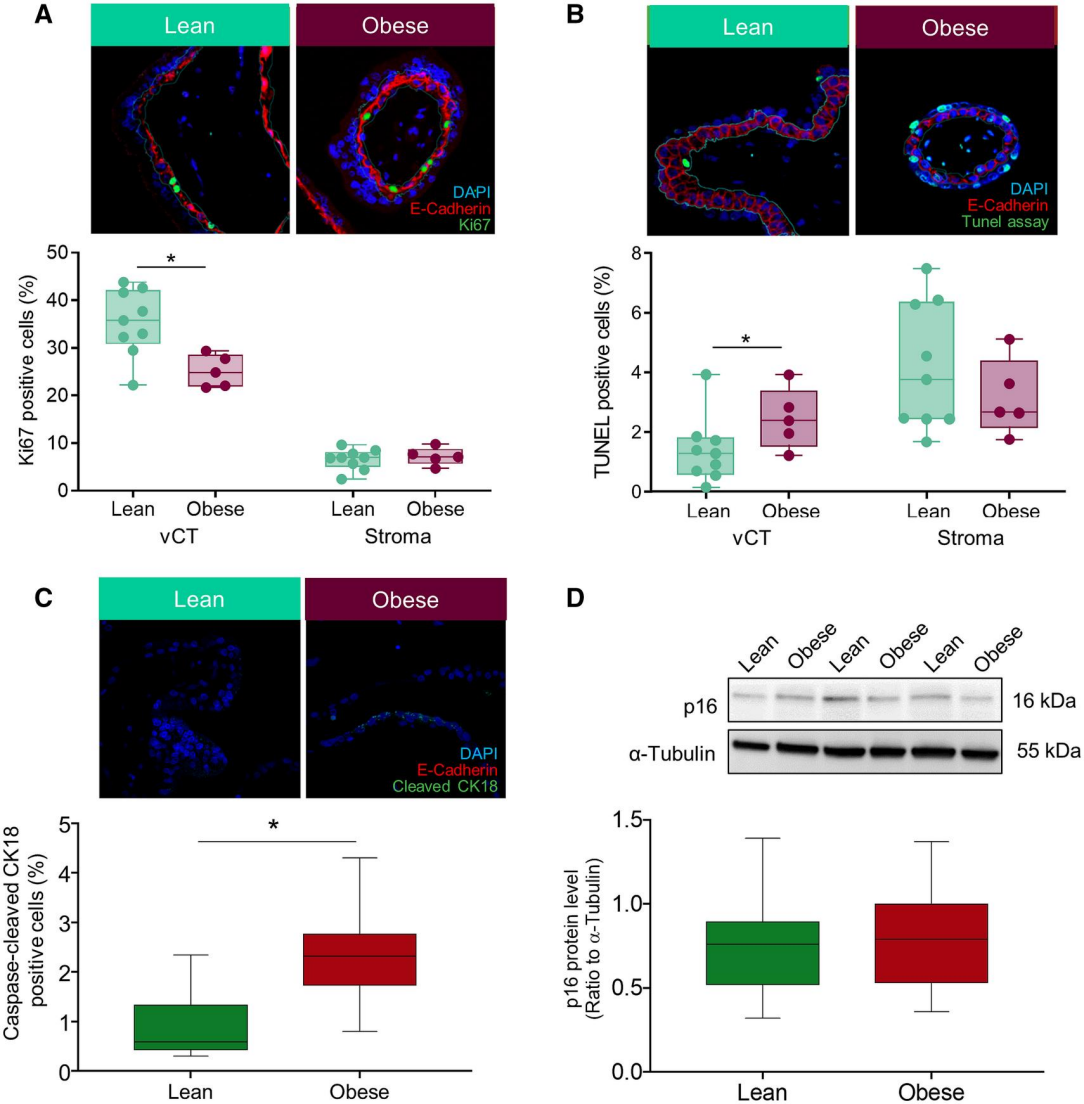
**Reduced proliferation and increased apoptosis characterize trophoblast turnover in first trimester (FT) obese placentas.** (**A**) Trophoblast proliferation was assessed by double immunofluorescence staining and semi-quantitative *in situ* analysis of the Ki67, proliferation marker and E-cadherin, trophoblast marker. (**B**) and (**C**) Apoptosis was assessed using the TUNEL assay and caspase-cleaved cytokeratin 18 (CK18), respectively. Ten randomly selected villi were captured per section. (**D**) Senescence marker p16 was determined in total protein of FT placental cell lysates by immunoblot and relative density ratio of each protein was calculated to α-tubulin. From (A) to (D), statistical analysis was performed using a multivariable linear regression analysis with BMI as categorical dependent variable and data were adjusted for gestational age, processing time, and fetal sex. Fetal sex did not influence the number of caspase-cleaved CK18 positive cells (*P* = 0.344) or the p16 protein level (*P* = 0.550) in multivariate ANOVA with fetal sex and BMI as categorical fixed variables and gestational age and processing time as covariates. Data are shown as mean ± SD, for (A) and (B) n (lean/obese) = 9/5, gestational week 5–7 and for (C) and (D), n (lean/obese) = 18/18, gestational week 5–12. *P < 0.05. BMI, body mass index; vCT, villous cytotrophoblasts.

Proliferation was reduced (−29.0%, *P* = 0.02) in samples from women with obesity (mean ± SD: 25.4 ± 3.6) relative to those from lean women (mean ± SD: 35.4 ± 7.0). The obesity-associated change was limited to vCTB, as stromal cells were unaffected (Fig. 6A). Reduced vCTB proliferation was paralleled by increased apoptosis (5.6-fold, *P* < 0.01), reflected by TUNEL positive cells, within the vCTB layer but not within the stroma of FT placental villi (Fig. 6B). These results were confirmed by immunofluorescence staining of caspase-cleaved CK18 showing increased vCTB staining levels (2.5-fold, *P* = 0.01) *in situ* in tissue from obese women (Fig. 6C). Protein levels of p16 did not differ in FT placental tissue between lean and obese women (Fig. 6D and Supplementary Fig. S4).

## Discussion

Adequate trophoblast proliferation, differentiation, and fusion are essential for successful placental development and function in early pregnancy (Aplin and Jones, 2021) and may be dysregulated in obesity (Hoch *et al.*, 2019) potentially compromising DNA integrity in the FT placenta. While obesity effects on extravillous CTB invasion suggest delayed spiral artery opening (St-Germain *et al.*, 2020) and other, predominantly metabolic, aspects of placental function in FT have been studied (Lassance *et al.*, 2015a,b; Bidne *et al.*, 2022; Rasool *et al.*, 2022), consequences on vCTB turnover have remained unknown and were the subject of this study.

The key findings are: (i) maternal obesity is associated with increased DNA damage in the FT vCTB, (ii) there is no evidence for oxidative stress-induced DNA damage, and (iii) DNA damage repair is insufficient to maintain vCTB in a proliferative state, hence, entailing apoptosis, but not senescence.

Obesity-induced placental DNA damage specifically localized to vCTBs without changes in STB or stromal compartments. This differs from the end of pregnancy, when abundant γH2AX immunostaining was observed in STB in other pregnancy pathologies (Cindrova-Davies *et al.*, 2018).

Obesity has been associated with oxidative stress and sustained low-grade inflammation, both classical inducers of DNA damage-mediated cell cycle arrest (Włodarczyk and Nowicka, 2019). In obese non-pregnant women, both oxidative and inflammatory stress correlate with DNA lesions and oxidative DNA damage (Al-Aubaidy and Jelinek, 2011; Włodarczyk *et al.*, 2018). In the present study, we found no evidence for obesity-associated oxidative DNA damage in the FT placenta. Neither classical stress markers, HSP70 and HO-1, nor levels of 8-OHdG in FT villous tissue and, specifically, in vCTBs were affected.

This is surprising since in FT, the placenta has low anti-oxidative defense systems, which gradually rise only at the end of the FT, making the placenta vulnerable to pro-oxidative forces (Jauniaux *et al.*, 2000). Classically, hyperglycemia is a key driver of oxidative stress through stimulating oxidative metabolism. However, obesity is not associated with maternal hyperglycemia in the FT (Bandres-Meriz *et al.*, 2020). Moreover, the low oxygen tension within the intervillous space in the early FT period and in particular because of suggested obesity-associated delay in spiral artery opening (St-Germain *et al.*, 2020) make NADPH oxidase an unlikely alternative source of oxidative stress (Raijmakers *et al.*, 2006). Thus, absence of oxidative DNA damage is compatible with our current knowledge of the early intrauterine environment both in general and in obesity.

Alternatively, a pro-inflammatory environment may induce DNA damage independent of oxidative stress (Higashimoto *et al.*, 2006). *In vitro* experiments using placental explants treated with a pro-inflammatory cytokine, TNF-α, at physiological oxygen tension resulted in changes of the DNA repair machinery genes with some overlap to *in situ* results, in which we compared total tissue from obese with that of lean women. This leads us to conjecture that obesity-related inflammatory stress enhances DNA damage by negatively affecting DNA repair machinery. Alternatively, or in combination, elevated γH2AX levels in vCTB, the trophoblast stem cells, may result from replicative stress, as observed in also rapidly dividing embryonic stem cells (Shaw *et al.*, 2009).

Despite higher ATM and ATR protein levels, phosphorylation of ATM/ATR substrates as well as the majority of selected downstream repair genes remained unaffected by maternal obesity. However, central DNA damage repair transducers (*HDAC1*, *PARP1*, *FEN1*, *BLM*, *XPA*, and *POLG*) were downregulated. These transducers are involved in SSB repair (BER) and DSB repair, e.g. non-homologous end joining (NHEJ), and homologous recombination (HR), pathways. Thus, elevated levels of DNA damage can also be the result of reduced repair capacity, not unprecedented, as it was also found outside pregnancy (Crespo-Orta *et al.*, 2023). Indeed, the results tend to indicate such a defect, not only because repair molecules are downregulated, but also because of the increased degree of apoptosis. This notion is supported by our recent finding of upregulated BRCA1 in FT placentas of obese pregnant women (Hoch *et al.*, 2020a), which, among many downstream effects, can also induce apoptosis (Takaoka and Miki, 2018). However, not all repair mechanisms may be equally affected as RAD52, a key protein for HR, is upregulated in FT placentas in obesity (Hoch *et al.*, 2020a).

Decreased vCTB proliferation and increased apoptosis with unaltered senescence in FT placentas from obese pregnant women are important findings and were replicated in a murine obesity model (Kim *et al.*, 2014). The implications may be a transiently reduced placental growth in the FT. This may entail consequences for further development of the feto-placental unit (Desoye and van Poppel, 2015), especially at the time when placental adaptive capacity is not fully developed yet (Desoye and Wells, 2021). Reduced placental growth in the early pregnancy period may also transiently limit fetal growth, which indeed was observed in a large cohort of obese women (Zhang *et al.*, 2018; van Poppel *et al.*, 2023). Future studies are needed to analyze in detail the complex processes leading to increased DNA damage and apoptosis in the FT human vCTB of pregnant obese women.

### Strengths and limitations

This study was only possible because of our unique FT cohort with placental tissue spanning a wide BMI and gestational age range. Smoking induces oxidative stress also in FT placenta (Hoch *et al.*, 2023). Using cotinine measurements combined with self-reported information, we carefully excluded smokers. We used an *in situ* method to quantify trophoblast-specific DNA damage thereby avoiding the cellular stress associated with the process of trophoblast isolation.

In the FT of human pregnancy, oxygen tension in the intervillous space and within the placenta gradually rises, a process that is critical for proper development and adequate trophoblast function (Jauniaux *et al.*, 2000; Burton *et al.*, 2010). Therefore, it was essential to include gestational age as *a priori* defined confounder in all statistical analyses. Initially, we established the finding of obesity-associated DNA damage for a narrow early time period in FT and then confirmed the obesity effect in the broad range of gestational ages in FT, although with a majority of samples obtained between 5 and 9 weeks. This preponderance of samples from early FT may explain the absence of increased oxidative stress and DNA damage markers in our cohort opposite to a study on late FT placenta tissue, i.e. from 8 to 13 weeks (Alanis *et al.*, 2012), highlighting the potential influence of oxygen tension in the intervillous space on placental oxidative stress. All tissue experiments were conducted at physiologically low oxygen tension (2.5% O_2_) to avoid potential hyperoxic effects of ambient oxygen (Shechter *et al.*, 2004).

We also have to acknowledge some limitations. Not all experiments could be conducted on the same tissue samples, because of the limited amount of tissue available precluding the full range of analyses. Thus, our strength in that we included a wide range of FT samples from weeks 5–12, was also a limitation as it made it impossible to identify specific periods in the FT, during which the placenta may have been particularly responsive to obesity. Absence of oxidative DNA lesions suggests most, if not all, DNA damage was in the nucleus rather than mitochondria (Włodarczyk and Nowicka, 2019), but this was not formally tested. Placental tissue was obtained from pregnancy terminations for psychosocial, i.e. non-medical, reasons. Thus, we do not know the outcome should pregnancies have continued, nor do we know whether there were genetic aberrations in the tissue.

Fetal sex modifies FT placental transcriptome (Gonzalez *et al.*, 2018) and many placental processes late in pregnancy (Myatt and Maloyan, 2016). The obesity-associated increase in the key outcome, i.e. DNA damage, assessed by alkaline COMET assay, did not differ between sexes similar to absence of sex effect on DNA damage outside pregnancy (Milic *et al.*, 2021). Nevertheless, we further tested for potential sex effects in some experiments due to limited sample size and did not find any sex dichotomy. Thus, our data do not suggest a profound sex effect, but future studies need to specifically assess the potential role of fetal sex for obesity-associated DNA damage, damage repair, and cytotrophoblast turnover.

## Conclusions

**Figure 7.**
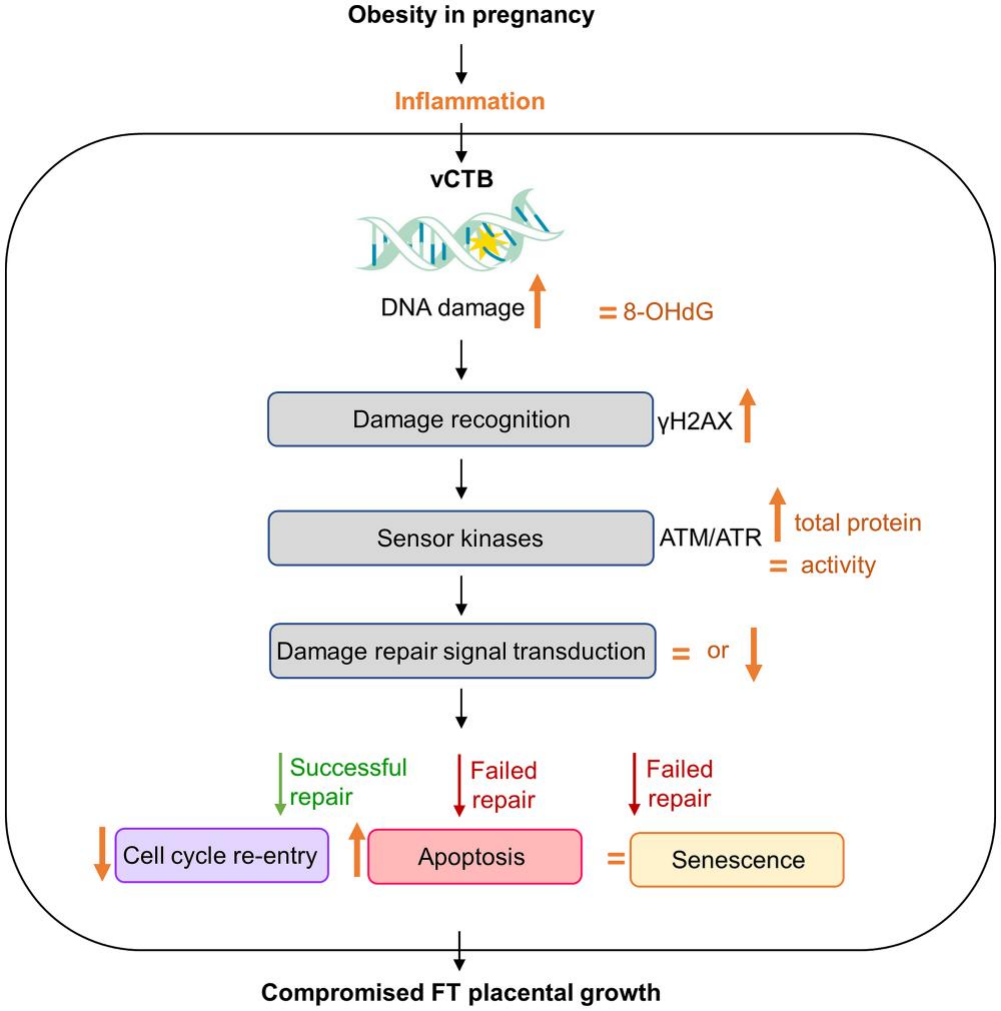
**Hypothetical schematic of maternal obesity effects on DNA damage and vCTB turnover in first trimester (FT) human pregnancies.** Obesity-associated meta-inflammation and not oxidative stress impairs AMT/ATR activation with dysregulated downstream DNA damage repair. This may lead to DNA damage accumulation that finally affects vCTB turnover in early obese placentas favoring apoptosis and compromising placental growth. ATM, ataxia telangiectasia mutated; ATR, ataxia telangiectasia and Rad3-related; vCTB, villous cytotrophoblasts; 8-OHdG, 8-hydroxydeoxyguanosine; γH2AX, H2A histone family member X.

Maternal obesity is associated with increased DNA damage in vCTB of FT human pregnancies. Inflammatory rather than oxidative stress appears to contribute to the damage. We envisage a scenario in which obesity-associated meta-inflammation impairs AMT/ATR activation with dysregulated downstream DNA damage repair. This may lead to DNA damage accumulation that finally affects vCTB turnover in obese placentas favoring apoptosis (Fig. 7). Ultimately, placental development may be altered, potentially ensuing consequences for later fetal growth and development.

## Supplementary Material

gaae027_Supplementary_Data

## Data Availability

The datasets generated during and/or analyzed during this study are available from the corresponding author upon reasonable request. No applicable sources were generated or analyzed during the current study.

## Author Biography

**Dr Silvija Tokic**: Dr Tokic is a cell biologist with a background in Biotechnology and a PhD in Molecular Medicine. Her research focuses on acquired and inborn metabolic disorders, both characterized by inflammation. She has been a group leader since 2022, and her lab works on characterization and implementation of patient-derived primary cell models to study disease pathophysiology and for use in preclinical drug testing. As of 2024, she has been the head of the research unit ‘Analytical Mass Spectrometry, Cell Biology and Biochemistry of Inborn Errors of Metabolism’ at the Department of Pediatrics and Adolescent Medicine at the Medical University of Graz.
