# Supplementary material for: Obesity-associated non-oxidative genotoxic stress alters trophoblast turnover in human first-trimester placentas
Source: Mol Hum Reprod. 2024 Aug 2;30(8):gaae027. doi: 10.1093/molehr/gaae027 (PMC11347397; doi:10.1093/molehr/gaae027)
Supplement: gaae027_Supplementary_Data [file gaae027_supplementary_data.pdf]

Supplementary Information

Obesity-associated non-oxidative genotoxic stress alters trophoblast turnover in human first trimester placentas

Denise Hoch, Alejandro Majali-Martinez, Julia Bandres-Meriz, Martina Bachbauer, Caroline Pöchlauer, Theresa Kaudela, Ezgi Eyluel Bankoglu, Helga Stopper, Andreas Glasner, Sylvie Hauguel-de Mouzon, Martin Gauster, Silvija Tokic and Gernot Desoye

Contents of Supplementary Information

Supplementary Table S1. Description of the study cohorts by experiment for the respective groups, lean vs obese.

Supplementary Figure S1. Full sized images of western blots shown in Figure 2E.

Supplementary Figure S2. Full sized images of western blots shown in Figure 3D.

Supplementary Figure S3. Full sized images of western blots shown in Figure 3E and F.

Supplementary Figure S4. Full sized images of western blots shown in Figure 6D.

Supplementary Table S1. Description of the study cohorts by experiment for the respective groups, lean vs obese.

Experiments	Characteristics	Maternal BMI (kg/m^2)	Gestational age (days)	Maternal age (years)	Fetal sex (%m)
**Alkaline COMET Assay *ex vivo* H_2O_2 FPG COMET Assay**	**Lean (n=10)**	21.3±1.4	68.0±12.5	25.1±7.8	50
	**Obese (n=14)**	34.4±2.9	51.4±10.3	28.1±6.2	75
	**p-value**	**> 0.0001**	**0.003**	0.320	
***in situ* γH2AX Ki67 TUNEL Assay**	**Lean (n=9)**	21.4±1.9	50.7±8.9	35.4±7.5	60
	**Obese (n=5)**	35.4±3.6	50.4±13.9	29.2±6.2	50
	**p-value**	**> 0.0001**	0.968	0.168	
**Nanostring**	**Lean (n=53)**	22.25±1.6	55.8±14.2	29.78±8.8	45
	**Obese (n=48)**	34.5±3.3	52.7±14.3	29.4±6.0	68
	**p-value**	**> 0.0001**	0.170	0.438	
**Immunoblot**	**Lean (n=18)**	21.6±1.1	51.9±13.9	30.8±6.2	59
	**Obese (n=18)**	34.3±3.1	51.7±13.2	29.2±6.1	55
	**p-value**	**> 0.0001**	0.972	0.443	
**8-OHdG ELISA**	**Lean (n=7)**	20.4±1.0	49.0±0.0	32.6±6.0	33
	**Obese (n=7)**	31.6±3.9	49.0±0.0	35.0±7.0	67
	**p-value**	**> 0.0001**	n.a.	0.531	
***in situ* 8-OHdG**	**Lean (n=12)**	23.0±1.8	62.3±15.6	31.2±7.5	0
	**Obese (n=9)**	34.9±3.7	50.7±16.2	30.3±7.0	0
	**p-value**	**> 0.0001**	0.114	0.798	

Following Shapiro-Wilk test for normality, data were compared using student's t-test. Data are given as mean ± SD. A multivariate analysis was performed to compare subjects´ characteristics between the experiments with experiment and group as dependent and maternal BMI, gestational age, maternal age and fetal sex as fixed variables. Only maternal BMI was statistically significant (p=0.009) whereas maternal age (p=0.226), gestational age (p=0.154) and fetal sex (p=0.732) were not significant. BMI, body mass index; m, male; γH2AX, H2A histone family member X; 8-OHdG, 8-hydroxydeoxyguanosine.

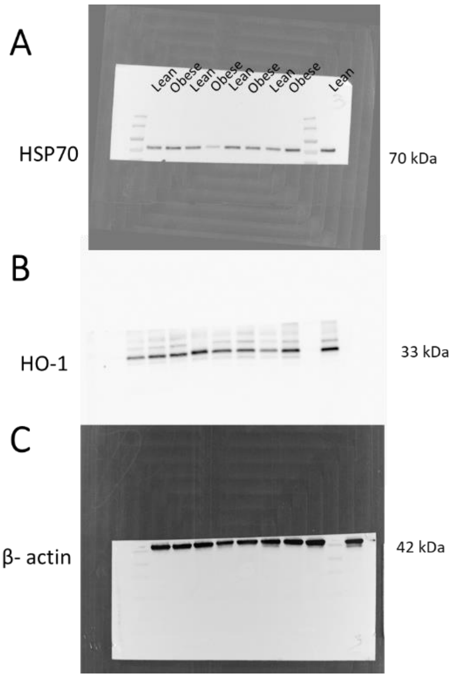

Supplementary Figure S1. Full sized images of western blots shown in Figure 2E. Due to limited first trimester tissue availability detection of A) HSP70 (70kDa) B) HO-1 (33kDa) and loading control (β-actin, 42kDa) were performed on the same membrane. Membrane was cut at 45kDa and upper part was used for detection of HSP70 while the lower part was used for HO-1 and β- actin. HSP70, heat shock protein 70; HO-1, heme-oxygenase 1.

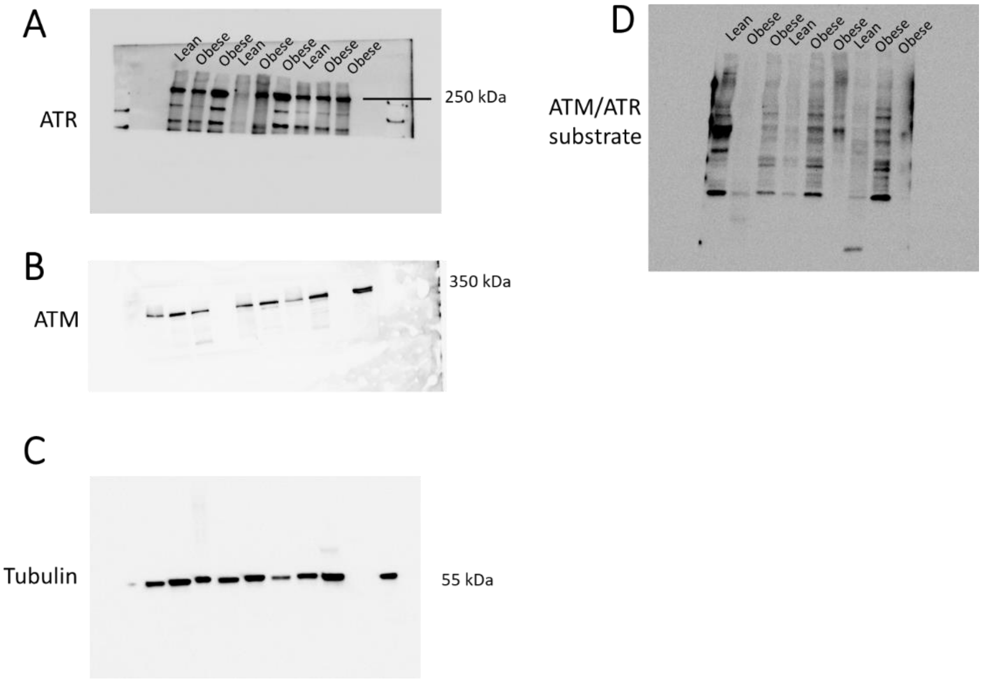

Supplementary Figure S2. Full sized images of western blots shown in Figure 3D. Due to limited first trimester tissue availability detection of A) ATR (250kDa) B) ATM (350 kDa) and loading control (α-tubulin, 55kDa) were performed on the same membrane. Membrane was cut at 100kDa and upper part was used for detection of ATM and ATR while the lower part was used for Tubulin. ATM, ataxia telangiectasia mutated; ATR, ataxia telangiectasia and Rad3-related

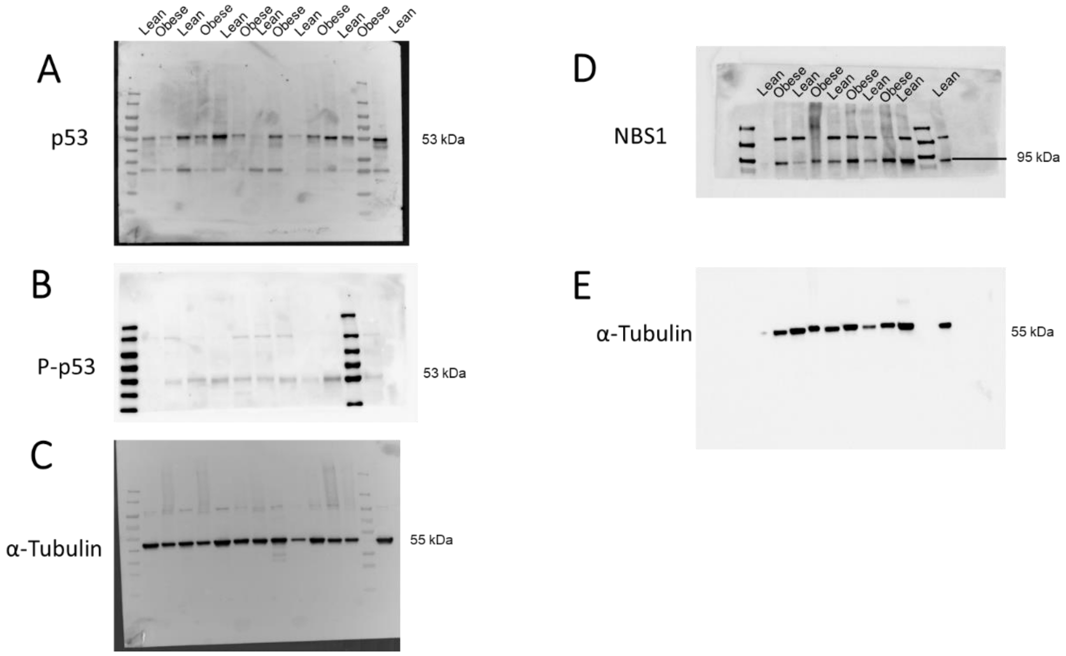

Supplementary Figure S3. Full sized images of western blots shown in Figure 3E and F. Detection of A) p53 (kDa), B) phosphorylated p53 and loading control α-tubulin (55kDa). Membranes used for ATM and ATR were stripped and used for detection of NBS1 D) and loading control α-tubulin (55kDa) E). ATM, ataxia telangiectasia mutated; ATR, ataxia telangiectasia and Rad3-related.

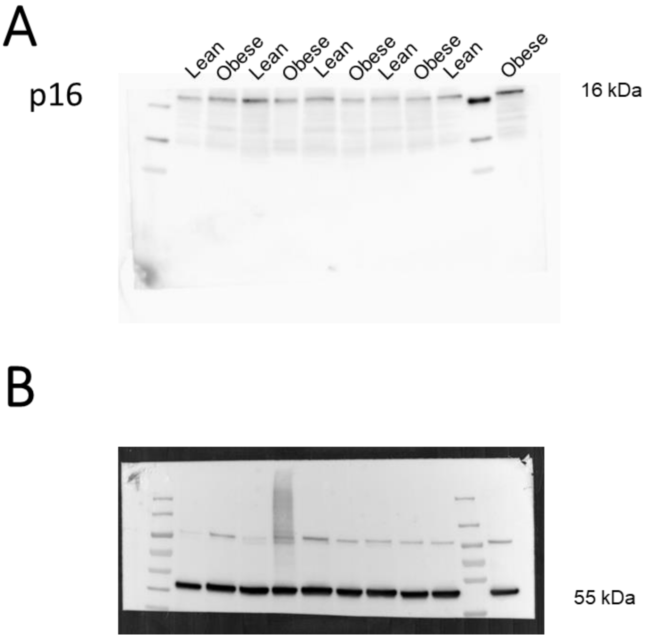

Supplementary Figure S4. Full sized images of western blots shown in Figure 6D. For detection of A) p16 (16 kDa) and loading control (α-tubulin, 55kDa) B) the membrane was cut at 40kDa and upper part was used for detection of p16 while the lower part was used for α-tubulin.
